# Opossum APOBEC1 is a DNA mutator with retrovirus and retroelement restriction activity

**DOI:** 10.1038/srep46719

**Published:** 2017-04-21

**Authors:** Terumasa Ikeda, Mayuko Shimoda, Diako Ebrahimi, John L. VandeBerg, Reuben S. Harris, Atsushi Koito, Kazuhiko Maeda

**Affiliations:** 1 Department of Retrovirology and Self-Defense, Faculty of Life Science, Kumamoto University, Kumamoto 860-8556, Japan; 2 Department of Biochemistry, Molecular Biology, and Biophysics, University of Minnesota, Minneapolis, Minnesota 55455, USA; 3 Institute for Molecular Virology, University of Minnesota, Minneapolis, Minnesota 55455, USA; 4 Masonic Cancer Center, University of Minnesota, Minneapolis, Minnesota 55455, USA; 5 Department of Immunology, Graduate School of Medical Science, Kumamoto University, Kumamoto 860-8556, Japan; 6 Laboratory of Host Defense, Research Institute for Microbial Diseases, Osaka University, Osaka 565-0871, Japan; 7 Laboratory of Host Defense, WPI Immunology Frontier Research Center (IFReC), Osaka University, Osaka 565-0871, Japan; 8 South Texas Diabetes and Obesity Institute, School of Medicine, The University of Texas Rio Grande Valley, Brownsville/Harlingen/Edinburg, Texas 78520, USA; 9 Howard Hughes Medical Institute, University of Minnesota, Minneapolis, Minnesota 55455, USA

## Abstract

APOBEC3s (A3s) are single-stranded DNA cytosine deaminases that provide innate immune defences against retroviruses and mobile elements. A3s are specific to eutherian mammals because no direct homologs exist at the syntenic genomic locus in metatherian (marsupial) or prototherian (monotreme) mammals. However, the A3s in these species have the likely evolutionary precursors, the antibody gene deaminase AID and the RNA/DNA editing enzyme APOBEC1 (A1). Here, we used cell culture-based assays to determine whether opossum A1 restricts the infectivity of retroviruses including human immunodeficiency virus type 1 (HIV-1) and the mobility of LTR/non-LTR retrotransposons. Opossum A1 partially inhibited HIV-1, as well as simian immunodeficiency virus (SIV), murine leukemia virus (MLV), and the retrotransposon MusD. The mechanism of inhibition required catalytic activity, except for human LINE1 (L1) restriction, which was deamination-independent. These results indicate that opossum A1 functions as an innate barrier to infection by retroviruses such as HIV-1, and controls LTR/non-LTR retrotransposition in marsupials.

Apolipoprotein B (*apoB*) mRNA editing enzyme catalytic subunit 1 (APOBEC1, A1) is a cytidine deaminase that physiologically edits *apoB* mRNA, which encodes a key protein involved in lipid transport^1^,^2^,^3^. A1 is a member of the AID/APOBEC family, which catalyses the conversion of cytosines to uracils within single-stranded DNA and RNA polynucleotides^4^,^5^. In this family, the APOBEC3 members (A3s; mainly human A3F and A3G) are well-characterised innate immune effector proteins that restrict the spread of retroviruses and LTR/non-LTR retrotransposons^4^,^6^,^7^,^8^.

Cell culture experiments have demonstrated that A1 enzymes are capable of inhibiting the replication of HIV-1, regardless of the presence of the HIV-1 Vif protein^9^,^10^,^11^,^12^. Analogous to A3 enzymes, A1 is encapsidated into assembling viral particles, and deaminates cytosines to uracils in nascent single-stranded viral cDNAs during reverse transcription^10^,^11^. This activity results in hallmark genomic strand G-to-A mutations. In addition, genomic strand C-to-T mutations are readily detectable indicating that A1 enzymes also have the capacity to edit viral genomic RNA^10^,^11^,^12^. Further cell culture studies have shown that A1 enzymes can suppress the infectivity of several viruses such as SIV, feline immunodeficiency virus (FIV), MLV, hepatitis B virus (HBV), and herpes simplex virus 1 (HSV-1)^11^,^13^,^14^,^15^,^16^ and the mobility of autonomous retrotransposons^17^,^18^. Although the biological functions of A1 in controlling viral infection and mobile elements *in vivo* remain unclear, the fact that proviral DNAs recovered from HIV-1-infected rabbit macrophages contained hallmarks of A1-mediated deamination suggests that A1 is a natural barrier to retroviral infection^19^. However, studies in *A1*-null mice did not support the function of A1 in blocking Friend retroviral infection *in vivo*^20^.

The grey, short-tailed opossum, *Monodelphis domestica*, belongs to the Metatheria, which is one of the three major groups of modern mammals and the closest relative of the Eutheria (Fig. S1a). Although eutherians encode at least one A3 family protein, there is no *A3*-like gene in the genomes of non-eutherian mammals (Fig. S1b), which was confirmed by the opossum genomic DNA sequence^21^, a pan-species Z1 PCR analysis^22^ and a BLAST search in this study (Fig. S1b). These observations suggest that the contribution of A1 to the innate immunity pathways of the marsupials could be greater than their contribution in eutherian mammals.

The opossum A1 is a protein of 235 amino acids that shares ~70% amino acid identity with those encoded by eutherian mammals (Fig. S2). Although the ability of this protein to edit *apoB* mRNA has previously been characterised^23^, whether it inhibits retroviruses including HIV-1 and LTR/non-LTR mobile elements remains to be determined. We addressed these questions using cell culture-based assays and found that opossum A1 from the small intestine is capable of restricting the infectivity of several retroviruses and the mobility of LTR/non-LTR retrotransposons. Taken together, our data indicate that the ability of A1 enzymes to protect the host genome from the invasion of foreign nucleic acids is conserved in marsupial and eutherian mammals.

## Results

### Opossum A1 is mutagenic in *Escherichia coli*

**Figure 1:**
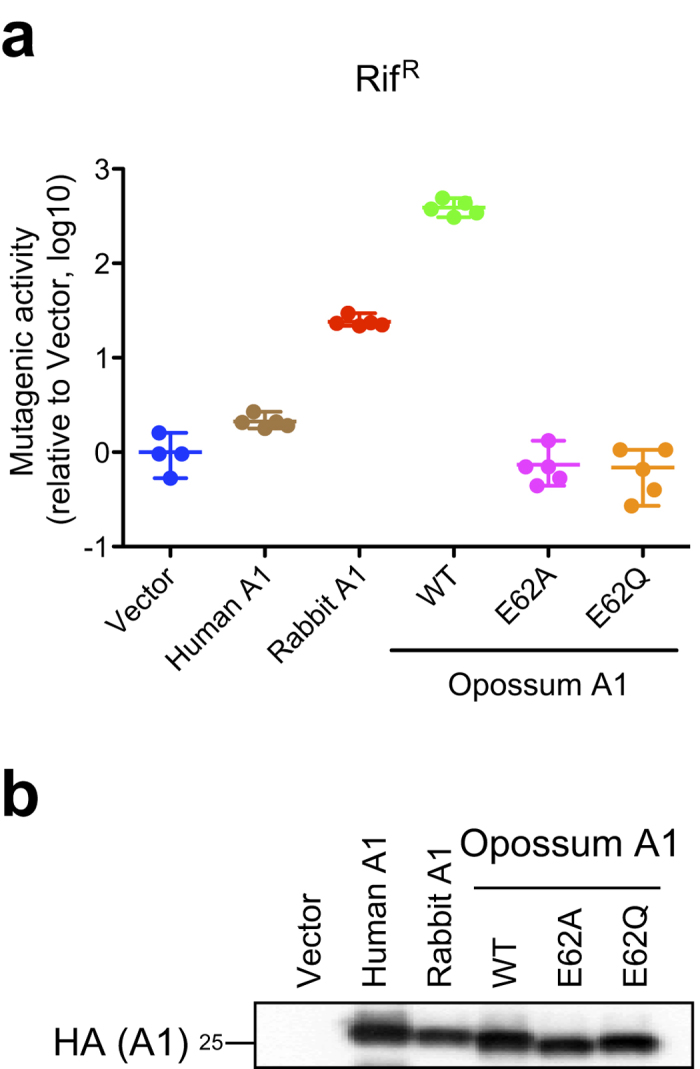
Mutagenic activity of opossum A1 in *E. coli*. (**a**) Dot plots reporting the mutagenic activity of the indicated constructs (Rif^R^ colonies per 10^7^ viable cells). Data are plotted relative to the vector control (n = 5, median with range). (**b**) Immunoblot of the indicated A1s expressed in bacteria.

Although the A1 protein encoded in marsupials can edit a synthetic *apoB* mRNA substrate^23^, its DNA-editing activity has not been determined. The bacterial mutator assay is widely used to evaluate the mutagenic activity of the AID/APOBEC family proteins^17^,^18^,^24^,^25^,^26^. In this assay, the expression of these proteins enhances the mutation of the bacterial RNA polymerase beta (*rpoB*) gene, which is reported as the frequency of rifampicin resistant (Rif^R^) colonies. Therefore, we used this system to measure the mutagenic activity of opossum A1 by counting the Rif^R^ mutant colonies that emerged during growth of multiple independent cultures (Fig. 1a). *A1* cDNA was isolated from opossum small intestine and cloned into an expression vector. Consistent with previous observations^12^,^18^, rabbit A1 increased the number of Rif^R^ colonies (24.1-fold relative to the vector control), whereas human A1 had only a slight effect (2.1-fold). Interestingly, the expression of wild-type (WT) opossum A1 in bacteria markedly enhanced the frequency of *rpoB* mutations (390-fold). However, this mutator phenotype was completely abrogated in catalytic mutant derivatives of opossum A1, E62A and E62Q (0.7-fold), even though they were expressed at levels similar to those of WT opossum A1 (Fig. 1b). These results demonstrate that opossum A1 has greater mutagenic activity than rabbit A1 in this *E. coli*-based mutation assay.

### Opossum A1 partially restricts HIV-1 infection

**Figure 2:**
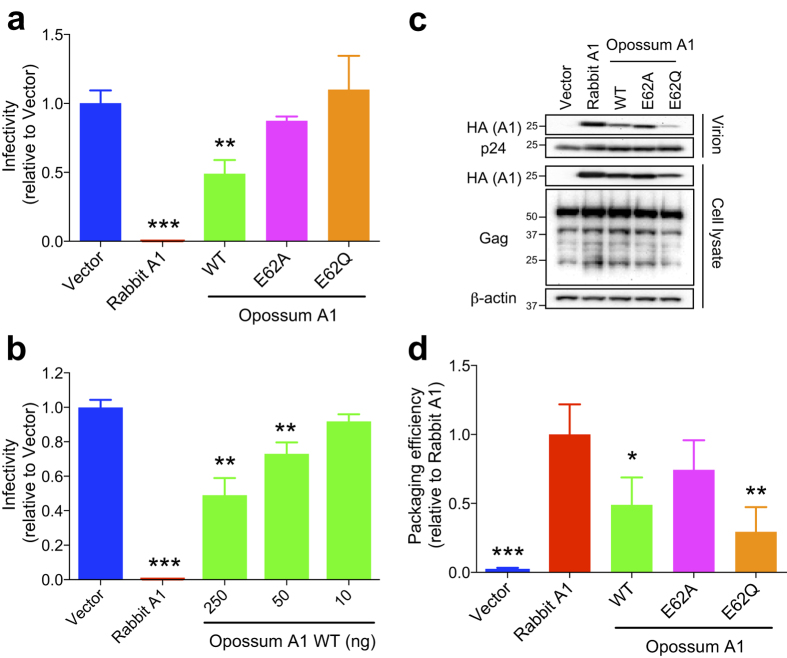
HIV-1 restriction by opossum A1. (**a**) Representative single-cycle assays with WT opossum A1 or deaminase-defective mutants (E62A or E62Q). Viral infectivity is plotted relative to vector control and is proportional to luciferase activity (n = 3, average +/− SD). *P* values are derived by comparisons with vector control data. (**b**) Representative single-cycle assays with varying concentrations of WT opossum A1 (10, 50 and 250 ng). Data represent the luciferase activity relative to vector control data (n = 3, average +/− SD; *P*-values as above). (**c**) Immunoblots of A1 in viral particles and cell lysates. A1 and HIV-1 Gag were detected using anti-HA anti-p24 antibodies, respectively. β-actin expression was used a loading control. (**d**) A histogram showing the relative packaging efficiency of each A1 construct. Band intensity of each HA-tagged A1 was normalised to the corresponding p24 level. Packaging efficiency is shown graphically relative to the packaging of rabbit A1 (n = 3, average +/− SD). *P* values represent comparisons with rabbit A1 data.

Several cell-based studies have demonstrated that the A1 proteins of rabbit and rodents inhibit HIV-1 replication independently of HIV-1 Vif expression^9^,^10^,^11^,^12^. To determine whether opossum A1 suppresses HIV-1 infectivity, we performed single-cycle HIV-1 infection assays in HEK293T cells. As expected, rabbit A1 caused a ~200-fold reduction in HIV-1 infectivity, with significant levels of A1 packaged into viral particles (Fig. 2). WT opossum A1 also inhibited HIV-1 replication compared with the vector control (~2-fold; *P* < 0.01), and the antiviral activity of opossum A1 was dose-dependent (Fig. 2a,b). The packaging level of the opossum protein was 2-fold lower than that of rabbit A1 (Fig. 2c,d). The antiviral activity of the WT opossum protein was abolished in the catalytic mutants, E62A and E62Q, regardless of the packaging level of the E62A mutant. These results indicate that opossum A1 partially restricts HIV-1 infectivity, and this restriction requires a catalytically active enzyme.

### Opossum A1 mainly localises to the nucleus

**Figure 3:**
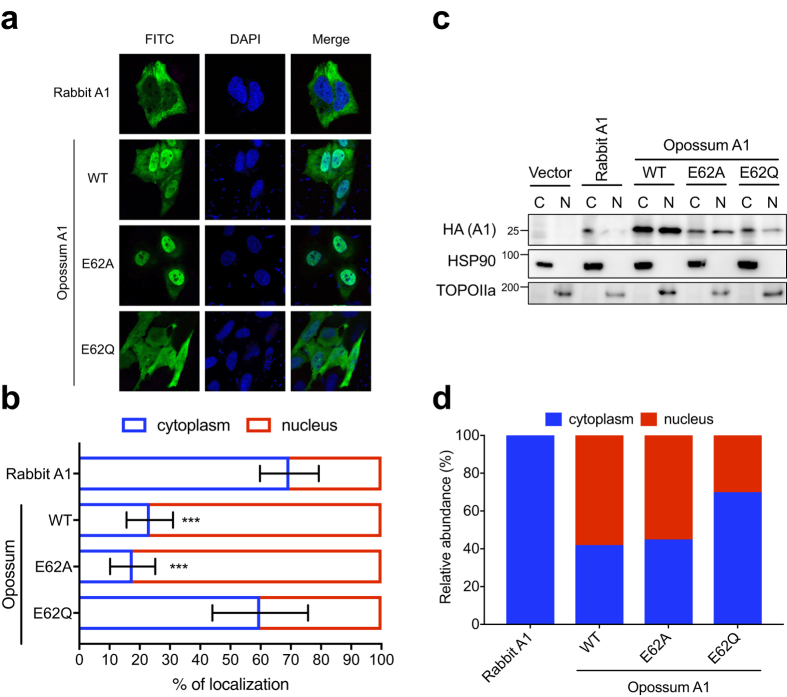
Subcellular localisation and expression levels of opossum A1 in HeLa cells. (**a**) Representative images showing HA-tagged A1s in green (FITC) and nuclei in blue (DAPI). (**b**) Quantification of the subcellular distribution of the indicated A1 constructs. The percentage nuclear and cytoplasmic localisation is indicated as the graphs (n = 20, average +/− SD). *P* values represent comparisons with rabbit A1. (**c**) Nuclear (N) and cytoplasmic (C) expression levels of A1s. Each cell fraction was blotted and probed with anti-HA, anti-HSP90, and anti-TOPOIIa antibodies. (**d**) Relative abundance of each A1 in nuclear or cytoplasmic fractions (percentage calculated based on combined intensity of C and N bands).

It has been demonstrated that the degree of A1 cytoplasmic localisation correlates with anti-HIV1 activity^12^. To assess this relationship here, we examined the subcellular localisation of opossum A1 in HeLa and HEK293T cells. The rabbit A1, which is the most active A1 protein against HIV-1^11^, was mainly distributed in the cytoplasm of both HeLa and HEK293T cells, where ~70% of the total signal was observed (Fig. 3a,b, S3). In contrast, ~80% of the total signal for WT opossum A1 was detected in the nuclei of both HeLa and HEK293T cells. The catalytic mutant E62A displayed the same subcellular localisation in HeLa and HEK293T cells as the WT protein. However, the E62Q mutation impaired the distribution of the protein, and the mutant protein was detected in the cytoplasm, implying aberrant localisation. These findings were confirmed by immunoblotting fractionated cell extracts (Fig. 3c,d). These results suggest that the moderate antiviral activity of opossum A1 correlates, at least in part, with lower steady state cytoplasmic levels of protein in cells.

### Opossum A1 partially restricts SIVs and MLV

**Figure 4:**
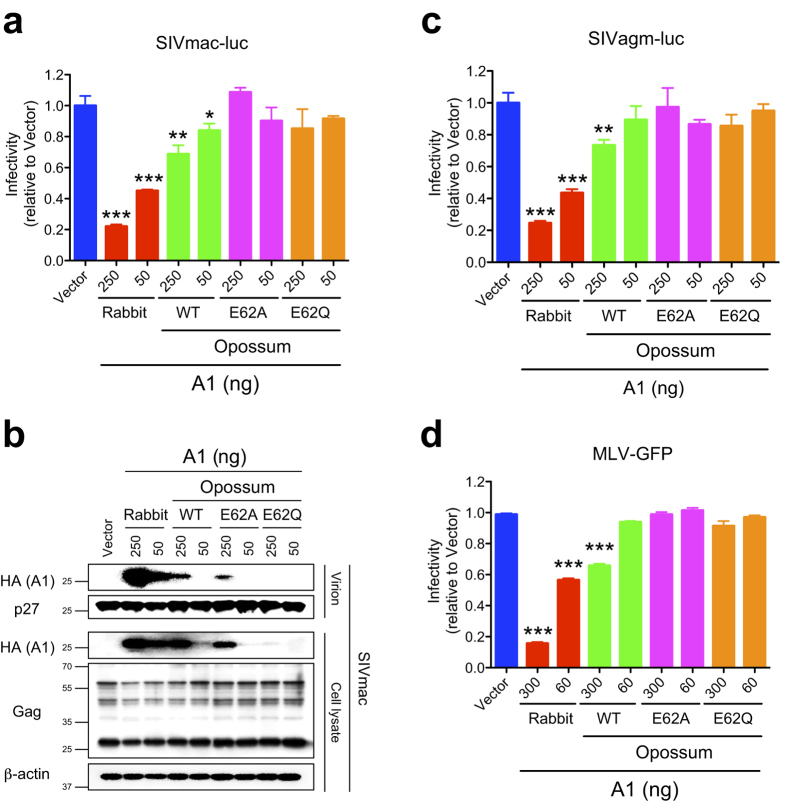
Inhibition of SIV and MLV replication by opossum A1. (**a**) Representative SIVmac single-cycle assays with varying concentrations (50 and 250 ng) of WT opossum A1 or deaminase-defective mutants (E62A or E62Q). Data represent the luciferase activity relative to vector control data (n = 3, average +/− SD with *P* values represented by asterisks). (**b**) Immunoblots of A1 in viral particles and cell lysates. A1 and SIVmac Gag were detected using anti-HA anti-p27 antibodies, respectively. β-actin was used a loading control. (**c**) Representative SIVagm single-cycle assays with varying concentrations (50 and 250 ng) of WT opossum A1 or deaminase-defective mutants. Data represent the luciferase activity relative to vector control data (n = 3, average +/− SD). *P* values represent comparisons with vector control data. (**d**) Representative MLV single-cycle assays with varying concentrations (60 and 300 ng) of WT opossum A1 or deaminase-defective mutants. Data represent the percentage of GFP+ cells relative to vector control data (n = 3, average +/− SD). *P* values represent comparisons with vector control data.

We have also demonstrated that the A1 proteins of rabbit and rodents inhibit the replication of several retroviruses including SIVs from rhesus macaque and African green monkey, and MLV^11^. To assess the capacity of opossum A1 to inhibit other retroviruses in addition to HIV-1, SIV and MLV pseudotyped viruses encoding a reporter gene were produced in the presence of opossum A1, and the effect of opossum A1 on retroviral replication was evaluated by infecting into HEK293T cells. Consistent with previous observation^11^, rabbit A1 inhibited the single cycle infection of SIVs from rhesus macaque and African green monkey (Fig. 4a to c), and MLV (Fig. 4d). A reduction in the infectivity of these viruses was also observed in the presence of opossum A1, and the degree of inhibition was similar to that of the anti-HIV-1 activity (see Fig. 2a,b versus Fig. 4a,c,d). The anti-retroviral activity against SIVs and MLV was similarly diminished by catalytic glutamate mutations (Fig. 4a,c,d), suggesting that catalytic activity is required for the restriction of these retroviruses by opossum A1. Thus, taken together with the HIV-1 data discussed above, these HEK293T-based experiments indicate that opossum A1 may have broad anti-retroviral activity.

### Opossum A1 inhibits human L1 retrotransposition using a deaminase-independent mechanism

**Figure 5:**
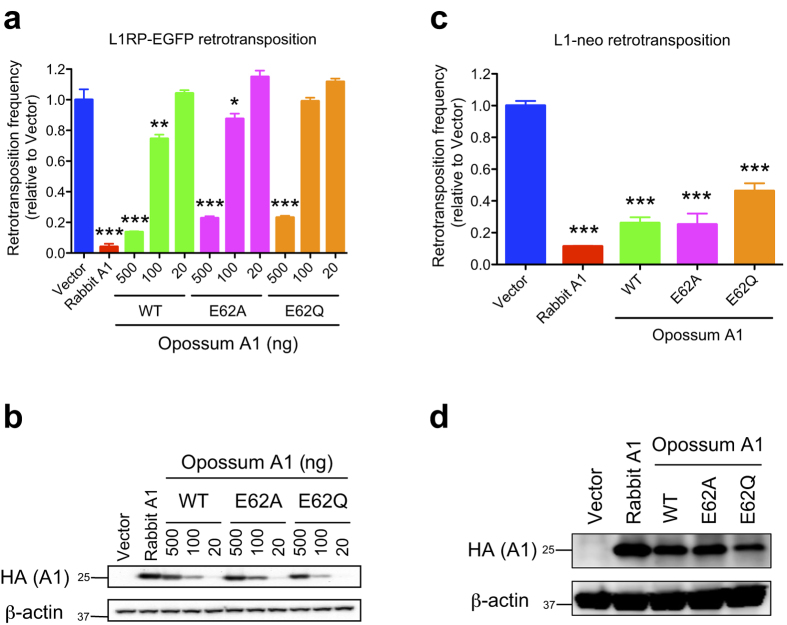
Restriction of L1-GFP retrotransposition by opossum A1. (**a**) Representative L1_RP_-GFP retrotransposition assays with varying concentrations (20, 100, or 500 ng) of WT opossum A1, or its catalytic mutants (E62A or E62Q). Retrotransposition frequency is presented relative to vector control data (n = 3, average +/− SD). *P* values represent comparisons with vector control data. (**b**) Immunoblotting of the indicated HA-tagged A1s expressed in HEK293T cells, with β-actin as the loading control. (**c**) A representative L1-neo retrotransposition assay with opossum A1 WT, or its catalytic mutants (E62A or E62Q). Retrotransposition frequency is presented relative to vector control data (n = 3, average +/− SD). *P* values represent comparisons with vector control data. (**d**) Immunoblotting of the indicated HA-tagged A1s expressed in HEK293T cells, with β-actin as the loading control.

We have previously shown that the A1 homologues of eutherian mammals use a deaminase-independent mechanism to inhibit L1 retrotransposition in human cell lines^18^. We tested the ability of opossum A1 to inhibit human L1 retrotransposition using enhanced green fluorescent protein (EGFP)-based retrotransposition assays in HEK293T cells^18^,^27^. Consistent with our previous observation^18^, rabbit A1 suppressed human L1 retrotransposition (~25-fold) (Fig. 5a,b). Similarly, human L1 retrotransposition was reduced ~7.7-fold in the presence of WT opossum A1 (500 ng), and the level of suppression was proportional to the amount of enzyme expression. This anti-L1 activity was not abolished by the inactivation of the deaminase activity of opossum A1 (Fig. 5a, E62A and E62Q). The anti-L1 activity of opossum A1 was also supported by similar results with neo-marked L1 retrotransposition assays in HeLa cells (Fig. 5c,d). These data indicate that opossum A1 inhibits human L1 retrotransposition independently of its catalytic activity.

### Opossum A1 suppresses MusD retrotransposition

**Figure 6:**
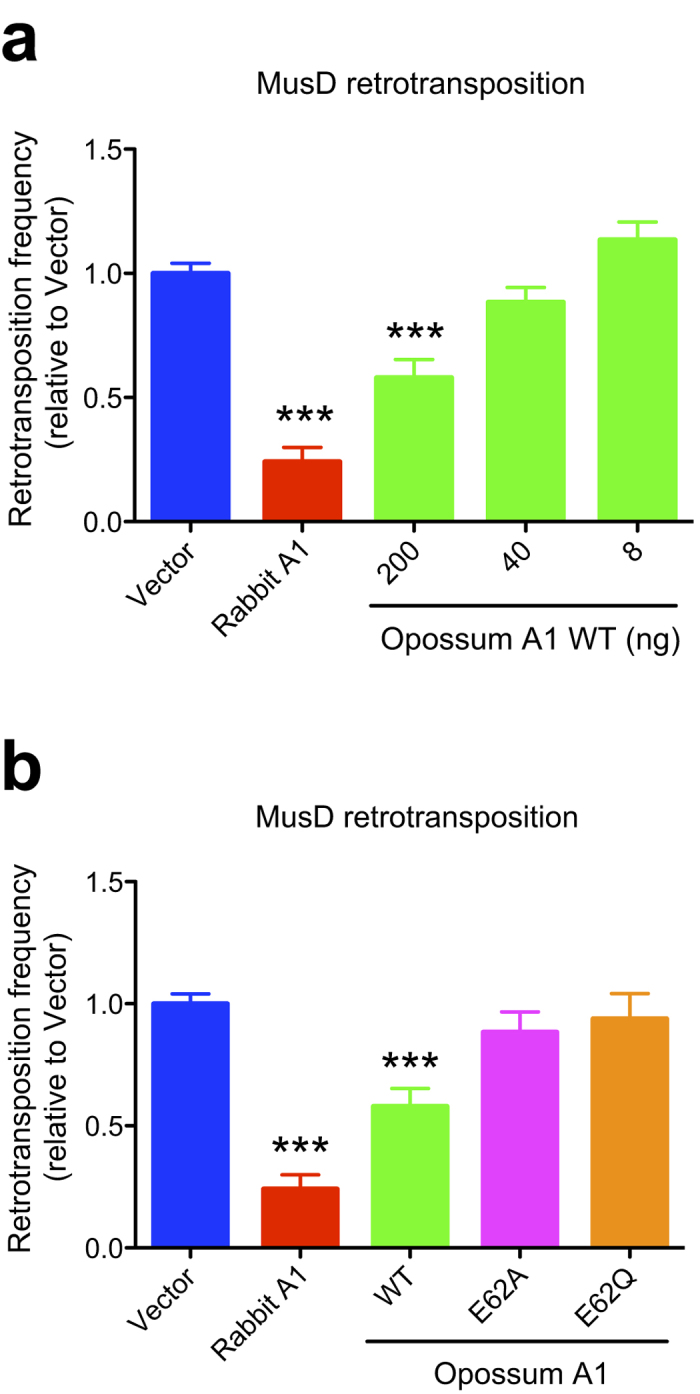
Suppression of MusD retrotransposition by opossum A1. (**a**) Representative MusD retrotransposition results in the presence of increasing amounts of WT opossum A1 (8, 40, or 200 ng). (**b**) MusD retrotransposition data with WT opossum A1 or deaminase-defective mutants (E62A and E62Q). Results are presented as retrotransposition frequencies relative to vector control data (n = 3, average +/− SD).

It has been shown that eutherian mammal A1 proteins also inhibit the mobility of LTR retrotransposons^18^. To test whether this activity is present in the opossum A1, we performed a neo-marked MusD retrotransposition assay in HeLa cells. As previously observed, rabbit A1 reduced MusD retrotransposition by ~4-fold (Fig. 6a). In the presence of opossum A1, a 2-fold reduction in the MusD retrotransposition frequency was observed, and this reduction was abolished when catalytic mutants E62A and E62Q were tested (Fig. 6b). These findings indicate that opossum A1 inhibits MusD retrotransposition in a deaminase-dependent manner.

### Opossum *A1* mRNA is widely expressed in primary tissues *in vivo*

**Figure 7:**
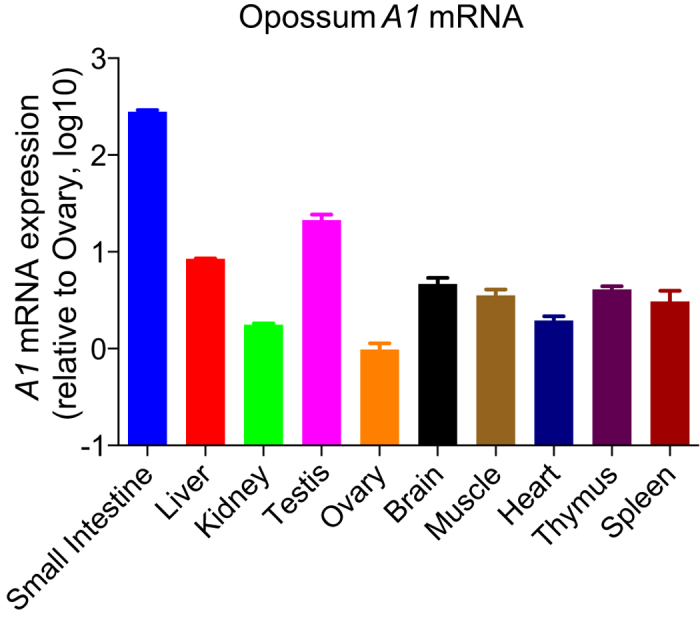
Expression levels of opossum *A1* mRNA in primary tissues. Representative opossum *A1* mRNA expression levels in primary tissues by RTqPCR. Data are presented as *A1* mRNA expression levels relative to those of the ovary normalized by endogenous expression levels of *gapdh* mRNA (n = 3, average +/− SD).

*A1* mRNAs from rabbit^18^, mouse^18^,^28^, and rat^28^,^29^ are broadly expressed in primary tissues such as the small intestine, spleen and gonads including both testis and ovary. In contrast, the expression of human *A1* mRNA is largely limited to gastrointestinal tissues^30^,^31^. We quantified the expression level of opossum *A1* mRNA in several primary opossum tissues. As anticipated, opossum *A1* mRNA was abundantly expressed in the small intestine (280-fold compared with the ovary), where it appears that A1 edits its *apoB* mRNA (Fig. 7). Interestingly, opossum *A1* mRNA was widely expressed in several tissues including the testis (22-fold) and lymphoid tissues (thymus, 4.1-fold; spleen, 3.3-fold) (Fig. 7). This expression pattern appears to be different from that of human A1, which was not reported to be expressed in the testis and lymphoid tissues^30^,^31^.

## Discussion

A3 enzymes are specific proteins of eutherian mammals that are involved in the innate immunity pathways directed against retroviruses and retrotransposons^4^,^5^,^6^,^7^,^8^. *A3* homologs do not appear to exist in non-eutherian mammals such as the opossum^21^,^22^ (Fig. S1b). In this study, we used a series of cell culture studies to examine whether opossum A1 has activity against retroviruses and LTR/non-LTR retrotransposons. Our results show that opossum A1 inhibits retrovirus replication and MusD retrotransposition in a deaminase-dependent manner (Figs 2, 4 and 6). This enzyme also suppresses human L1 retrotransposition in a deaminase-independent manner (Fig. 5). These results suggest that A1 plays role in protecting marsupial host genomes from parasitic genetic elements.

Despite potent mutagenic activity in bacteria (Fig. 1), opossum A1 showed modest HIV-1 restriction activity in HEK293T cells (Fig. 2). This relatively weak phenotype may reflect the predominantly nuclear localisation of opossum A1 in comparison to the more potent and predominantly cytoplasmic rabbit A1 enzyme (Figs 3 and S3). These results are consistent with our prior studies reporting that the degree of nuclear localisation of A1 correlates inversely with its antiviral activity^12^. It is likely that the nuclear distribution of opossum A1 prevents interaction with the Gag nucleocapsid protein, which in turn reduces encapsidation into virions. Alternatively, the physiological localisation of opossum A1 could differ from its localisation when overexpressed experimentally because endogenous A1 levels are below the limit of detection with currently available antibodies^32^. Furthermore, the nuclear localisation of A1 enzymes from different species appears to be influenced by cell-type^33^, suggesting that cellular factors regulate A1 localisation. A1s are expected to be more active against HIV-1 when they are in the cytoplasm. It must be noted that a chimeric human A1 fused to the rabbit homologue redistributed to the cytoplasm from the nucleus, was packaged more efficiently, and subsequently inhibited HIV-1 infectivity more effectively than the original human A1 protein^12^. Further studies, including the development of more sensitive antibodies and the identification of cellular factors that regulate the subcellular localisation of A1 will clarify the correlation between the subcellular localisation of A1 and its anti-HIV-1 activity.

Our cell-based assays did not use opossum cells and, to our knowledge, vectors have yet to be developed for opossum viruses and mobile elements. Nevertheless, the results presented here suggest that A1 likely functions as an innate barrier to infection by retroviruses and controls LTR/non-LTR retrotransposition in marsupials. First, opossum A1 possesses restriction activity against several different retroviruses (Figs 2 and 4) and LTR/non-LTR retroelements (Figs 5 and 6). Second, ~50% of the opossum genome is composed of repetitive elements (LINEs: ~29%, SINEs: ~10%, endogenous retroviruses: ~11% and DNA transposons: ~2%)^21^. Of note, the proportion of LINEs in the opossum genome is approximately 1.5 times higher than that in human and mouse genomes (~20% LINE). The greater anti-L1 activity of opossum A1 in comparison to anti-retroviral activity might be associated with the relatively higher proportion of LINEs in the opossum genome. Third, *A1* mRNA is expressed in many different opossum tissues (Fig. 7), which is distinct from the gastrointestinal expression profile of *A1* in human tissues^30^,^31^. This broad expression profile suggests that opossum A1 has additional functions besides editing of *apoB* mRNA. Future investigations should include the development of an infectious molecular clone using viruses that naturally infect opossum and assay systems using opossum cells, to determine whether opossum A1 is indeed a *bona fide* anti-viral restriction factor.

One of the common functions among AID/APOBEC family proteins is restriction of non-LTR retrotransposon, L1, using a mechanism that is independent of enzymatic activity^17^,^18^,^34^,^35^,^36^,^37^,^38^. However, it has been proposed that the mechanism by which L1 retrotransposition is suppressed by A1 is different from the deamination-independent mechanisms used by A3 enzymes^17^. In contrast to human A3s that are able to restrict human L1 retrotransposition even in the absence of a L1-encoded ORF1 protein^39^, A1s from human and lizard require the binding to L1 ORF1 protein for the inhibition of human L1 retrotransposition^17^. Interestingly, human and mouse ORF1 proteins form ribonucleoprotein (RNP) complexes with L1 RNA^40^,^41^, and the targeting of the L1 RNP complexes into cytoplasmic foci like stress granules is likely to control L1 retrotransposition^42^,^43^. It is noteworthy that human L1 RNA is found in A1 RNP complexes when they are cotransfected into HEK293T cells^18^. These observations suggest that the formation of A1 RNP complexes could sequester L1 RNA and/or ORF1 protein from its appropriate trafficking and translation, and ultimately could interfere with its retrotransposition into genome. This is also in contrast to A3s that are not necessary to form intracellular RNP complexes for L1 inhibition^36^. It is unclear what steps of L1 replication the A1 and A3 enzymes affect using deaminase-independent mechanisms. Elucidation of this deamination-independent repressive activity of APOBECs on L1 retrotransposition may provide important insights into the continuous arms race between viruses and their hosts.

It has been demonstrated that A1s from amniotes, including eutherian mammals, inhibit L1 retrotransposition *in vitro*^17^,^18^, and that these A1s also function as DNA mutators^18^,^44^. However, although A1 is expressed in the lizard, no deamination at the site corresponding to mammalian *apo*B mRNA editing has been observed^44^, suggesting that RNA editing may not be the ancestral function of A1. Because opossum A1 has RNA editing activity against *apoB* mRNA^23^, it appears that at least the A1s of mammals acquired the ability to edit *apoB* mRNA after the divergence of this group from the common ancestor they shared with other vertebrates (i.e., amphibians and birds). Therefore, the most likely original function of A1 was probably DNA editing and protecting cells against mobile elements, because the A1s of amniotes, including mammals, maintain anti-L1 activity *in vitro*^17^,^18^. Although several studies have suggested that non-human A1s (*e.g.*, rabbit A1) may be involved in innate immune pathways^11^,^13^,^14^,^16^,^18^,^19^, it seems that the function of human A1 is limited to editing *apoB* mRNA in the small intestine^30^, and that the functions of A1 in innate immunity have been taken over by the expansion of A3s in the primate lineages such as in humans. Further studies are needed to shed light on the evolutionary history of RNA editing by the A1 deaminases and on their ancestral and physiological functions other than *apoB* mRNA editing.

## Methods

### Cells and antibodies

HEK293T, and HeLa cells were maintained in Dulbecco’s modified Eagle’s medium supplemented with 10% bovine fetal serum (Gibco, Life Technologies). The anti-p24 capsid (CA) antibody used has been described previously^45^. The anti-SIVmac p27 antibody (55-2F12, #1610)^46^ was obtained from the NIH AIDS Reagent program. Antibodies against hemagglutinin (HA; C29F4, Cell Signaling Technologies, and HA.11, Covance), HSP90 (AC88, Stressgen), TOPOIIa (#4733, Cell Signaling Technologies), and β-actin (AC-74, Sigma) were commercially available.

### DNA constructs

Vectors expressing Vif-proficient HIV-1, SIVmac and SIVagm proviral DNAs (pNL4-3 Luc E^−^R^−^, SIVmac Luc E^−^R^−^ and SIVagm Luc E^−^R^−^, kindly provided by N.R. Landau, New York University)^47^, EGFP-based human L1 (pL1_RP_-EGFP, kindly provided by E.T. Luning Prak, University of Pennsylvania)^27^, *neo*^*R*^-gene-marked human L1 (pCEP4/L1mneoI/ColE1, kindly provided by N. Gilbert, Institut de Génétique Humaine, CNRS)^48^, and *neo*^*R*^-gene-marked murine MusD (pCMV L1Mus-6DneoTNF, kindly provided by T. Heidmann, Institut de Cancérologie Gustave Roussy)^49^ have been described elsewhere. The generation of a C-terminal HA-tagged rabbit A1 expression plasmid and bacterial expression plasmids encoding HA-tagged human and rabbit A1s has been described previously^18^.

### Cloning of opossum *A1* cDNA

Production, maintenance and experimental manipulations of the animals were approved by the Institutional Animal Care and Use Committee of the Texas Biomedical Research Institute (John L. VandeBerg; previous affiliation). All procedures were carried out in accordance with the approved guidelines. Small intestine has been removed aseptically from euthanized *Monodelphis Domestica* (opossum). Total RNA was isolated using TRIzol reagent (Invitrogen), and then treated with DNase I (Takara). cDNA encoding the opossum *A1* gene was synthesised using the High Capacity cDNA Reverse Transcription Kit (Applied Biosystems) with a primer set designed so that a single HA epitope tag was attached to the C-terminus of the protein as previously described^23^. (5′-NNNNNGATATCGAAGCCATGAATTCTAAGACAGGTCCA-3′ and 5′-NGCGGCCGCTCAAGCGTAATCTGGAACATCGTATGGGTATCTCCAGGTCACAAATGGCTGG-3′, Restriction enzyme sites are underlined). The amplified fragment was cloned into the pCR-Blunt vector (Invitrogen) and sequenced. The amplified product was then inserted into the *Eco*RV and *Not*I sites of the pCAGGS expression vector and sequenced. The nucleotide sequence of an open reading frame encoding opossum A1 was identical to that enrolled in GenBank (NM001032982). The catalytic mutants of the opossum A1 were constructed using the oligonucleotide primers. E62A: 5′-TCTCAACATGCTGCAATCAACTTCATG-3′ and 5′-CATGAAGTTGATTGCAGCATGTTGAGA-3′. E62Q: 5′-TCTCAACATGCTCAAATCAACTTCATGG-3′ and 5′-CCATGAAGTTGATTTGAGCATGTTGAGA-3′. The amplified products were inserted into the pCAGGS vector at the *Eco*RV and *Not*I sites and sequenced. Bacterial expression plasmids encoding C-terminal HA-tagged opossum A1 and its catalytic mutants (E62A and E62Q) were created with the following primer set: 5′-NNNNCTCGAGATGAATTCTAAGACAGGTCCATCAGTAGGAGA-3′ and 5′-NNNATGCATTCAAGCGTAATCTGGAACATCGTATGGGTA-3′. The amplified products were then cloned into the pTrcHis A vector (Invitrogen) with restriction enzymes *Xho*I and *Nsi*I and sequenced.

### Sequence alignment

The amino acid sequences of full-length mammalian A1s were aligned as previously described^11^. The GenBank accession numbers of the A1 sequences used for this comparison are: human (NM001644), rabbit (U10695), rat (MN012907), mouse (NM031159), and opossum (NM001032982).

### Immunoblot analysis

Cell and virion lysates were resolved with sodium dodecyl sulphate (SDS) polyacrylamide gel electrophoresis (PAGE), transferred to a polyvinylidene difluoride membrane (Millipore), and blocked with 4% milk in phosphate-buffered saline (PBS) containing 0.1% Tween 20. The membranes were then incubated with a primary antibody, a biotin-conjugated secondary anti-mouse or anti-rabbit IgG antibody (Sigma) and streptavidin-conjugated horseradish peroxidase (Sigma). The signals were visualised with Chemi-Lumi One (Nacalai Tesque) and a VersaDoc 5000 Imager (Bio-Rad).

### Subcellular localisation

An immunofluorescence analysis was performed as previously reported^12^,^18^. Briefly, 2 × 10^4^ HEK293T or HeLa cells were plated in each well of an eight-well Lab-Tek Chamber Slide (Nalge Nunc International). The cells were transfected with the pCAGGS expression vector (500 ng) encoding either HA-tagged rabbit A1, opossum A1, or its catalytic mutants (E62A or E62Q), using FuGENE HD (Roche). After 24 h, the transfected cells were fixed with 4% formaldehyde in PBS for 30 min, and permeabilised with 0.1% Triton X-100 in PBS. The cells were then treated with 0.1 M glycine/PBS to quench the reaction and blocked with 0.3% bovine serum albumin (BSA)/PBS. To stain A1, the cells on coverslips were incubated with an anti-HA antibody (HA.11; 1:1000 dilution) in 0.3% BSA/PBS in a humid chamber at 37 °C for 1 h, and then with fluorescein isothiocyanate (FITC)-conjugated goat anti-mouse IgG antibody (Sigma; 1:300)/0.3% BSA/PBS for 1 h. The cells were then treated with 1 μg/mL 4,6-diamidino-2-phenylindole staining (DAPI; Invitrogen) for 5 min. The coverslips were mounted with Fluorescent Mounting Medium (Dako). Fluorescence was visualised with a Zeiss LSM 700 laser-scanning confocal microscope. The images were captured with IPLab and processed with the Adobe PhotoShop 4.0 software. The fluorescence intensity of A1 in the cytoplasm was calculated as previously described^12^. Briefly, the total FITC signal intensity of 20 cells for each A1 was measured using Image J software. The intensity of FITC signal overlapped with the portion of DAPI staining was measured to calculate the proportion of nuclear distribution of A1. Then, the proportion of cytoplasmic distribution of each A1 was calculated by subtracting the nuclear FITC signal from the total FITC signal. The densitometric analysis of the blotting bands was quantified using an image analysis system.

### Preparation of nuclear and cytoplasmic extracts

The nuclear and cytoplasmic extracts from HEK293T were prepared with the Subcellular Protein Fractionation Kit for Cultured Cells (Thermo Fisher Scientific), according to the manufacturer’s protocol.

### Bacterial mutator assay

The generation of bacterial expression plasmids encoding cDNAs encoding HA-epitope-tagged opossum A1 and its derivatives is described above. The *Escherichia coli*–based DNA mutation assay was performed as described previously^12^,^18^. The uracil DNA glycosylase (*ung*)-deficient *E. coli* strain BW310 was transformed with the parental pTrcHisA plasmid or vectors encoding the various A1 cDNAs. Twenty colonies of the transformed bacteria, selected on plates containing ampicillin, were cultured overnight at 37 °C in 2 mL of LB medium containing ampicillin and 1 mM IPTG. The saturated cultures (100 μL) were then plated on LB agar containing 100 μg/mL rifampicin. The total number of Rif^R^ colonies per plate was counted after 24 h. The appropriate dilution of viable cells was plated onto an LB plate containing ampicillin and the mutation frequencies were calculated as Rif^R^ colonies per viable cell. To verify protein expression, 100 μL of each saturated IPTG-induced culture was lysed and subjected to immunoblotting, as described above.

### Single-cycle infection assays

HEK293T cells (2.5 × 10^5^) were cotransfected with 0.75 μg of Vif-proficient NL4-3 Luc E^−^R^−^ proviral DNA construct, together with 0.5 μg of pVSV-G vector and 0.25 μg of the control vector or one of several expression vectors encoding C-terminal HA-epitope-tagged A1 protein supplemented with the appropriate concentration of the control vector, using Effectene^®^ (Qiagen). For SIVs, 0.75 μg of Vif-proficient SIVmac or SIVagm Luc E^−^R^−^ proviral DNA construct was cotransfected into HEK293T cells, with 0.5 μg of pVSV-G vector and 0.25 μg of the control vector or HA-tagged A1 expression vector. MLV-GFP virus stock was produced as previously described^50^,^51^. HEK293T cells were cotransfected with 0.3 μg of MLV genome expressing a GFP reporter M3P-GFP, together with 0.3 μg of MLV packaging construct pMD-MLV-OGP, 0.1 μg of pVSV-G and 0.3 μg of the control vector or HA-tagged A1 expression vector. After 48 h, the virus-containing supernatants were filtered with 0.45 μm filters and frozen as aliquots. The p24 or p27 content of the viruses was determined with a HIV-1 p24 or SIV p27 antigen ELISA kit (ZeptoMetrix). Fresh target HEK293T cells were infected with the equivalent amounts of the luciferase reporter viruses and after 48 h, the intracellular luciferase activity of the infected cells was measured, as previously described^11^,^12^. Infectivity of MLV-GFP virus was analysed by flow cytometery. To assess the packaging of the A1 proteins into viral particles, the virus-containing supernatants were spun through a 20% sucrose cushion and the viral pellets were solubilised in 2× SDS sample buffer. Equivalent amounts of p24 or p27 antigen from each solubilised sample were analysed with immunoblotting, as described above. Band intensities were analysed with the ImageJ software.

### L1 retrotransposition assays

The EGFP-based human L1 retrotransposition assay was performed as previously described^18^. Briefly, 3 × 10^5^ HEK293T cells were cotransfected with 1.5 μg of pL1_RP_-EGFP or the pIRESpuro vector (Clontech) and 0.5 μg of the control vector or the respective A1 expression vector with the appropriate concentration of a control vector with Effectene. At 24 h posttransfection, the cells were selected with 1.0 μg/mL puromycin and 7-9 days after selection, the EGFP expression resulting from retrotransposition was measured with flow cytometry. The *neo*^*R*^-gene-marked human L1 retrotransposition assay was performed by cotransfecting HeLa cells (5 × 10^5^ cells) with 0.4 μg of the respective A1 expression plasmid and 1.2 μg of the human L1-neo reporter vector together with 0.4 μg of pIRES-EGFP (Clontech) as the transfection control, using FuGENE, as previously described^18^. After 72 h, 5 × 10^5^ cells were plated onto 100 mm dishes and selected with 0.75 mg/mL G418. At 12–14 days after selection, the resultant G418^R^ colonies were stained with crystal violet (Sigma) and counted. The retrotransposition frequency was calculated as the number of G418^R^ colonies/transfection efficiency (percentage of GFP^+^ cells).

### MusD retrotransposition assay

The murine MusD retrotransposition assay was performed as previously described^18^. Briefly, 5 × 10^5^ HeLa cells were cotransfected with 0.2 μg of the control vector or the respective A1 expression plasmid supplemented with the appropriate concentration of the control vector, together with 0.6 μg of the *neo*^*r*^-based murine MusD reporter vector together with 0.4 μg of pIRES-EGFP, using FuGENE. After 72 h, 5 × 10^5^ cells were reseeded onto 100 mm dishes for G418 (1.0 mg/mL) selection, and the resultant G418^R^ colonies were counted. The retrotransposition frequency was calculated as described above.

### Quantitation of opossum *A1* transcripts *in vivo*

cDNAs from opossum primary tissues were synthesized using the High Capacity cDNA Reverse Transcription Kit. Amplification was performed using the following primers: *A1*, 5′-CGCGTCCACCTGATTC-3′ and 5′-CGCGTCCACCTGATTC-3′. *gapdh*, 5′-CGCGTCCACCTGATTC-3′ and 5′-CGCGTCCACCTGATTC-3′. PCR amplification conditions were 95 °C for 10 min, followed by 50 cycles of 95 °C for 30 s, 53 °C for 20 s, and 68 °C for 30 s. Quantitation was performed with SYBR Green PCR master mix (Applied Biosystems) using a Realplex^2^ Mastercycler EPgradient S (Eppendorf). Expression levels of target cDNA were normalized to the amount of endogenous mRNA of *gapdh*.

### Statistical analysis

The statistical analysis was performed with Student’s *t* test. All data are the means and standard deviations (SDs) of three experiments, unless otherwise stated. *P < 0.05, **P < 0.01, ***P < 0.001.

## Additional Information

**How to cite this article:** Ikeda, T. *et al*. Opossum APOBEC1 is a DNA mutator with retrovirus and retroelement restriction activity. *Sci. Rep.* **7**, 46719; doi: 10.1038/srep46719 (2017).

**Publisher's note:** Springer Nature remains neutral with regard to jurisdictional claims in published maps and institutional affiliations.

## Supplementary Material

Supplementary Information
