# Supplementary material for: Opossum APOBEC1 is a DNA mutator with retrovirus and retroelement restriction activity
Source: Sci Rep. 2017 Apr 21;7:46719. doi: 10.1038/srep46719 (PMC5399452; doi:10.1038/srep46719)
Supplement: Supplementary Information [file srep46719-s1.pdf]

Supplementary Online Data

Opossum APOBEC1 is a DNA mutator with retrovirus and retroelement restriction activity

Terumasa Ikeda[1,2,3,4,*], Mayuko Shimoda[5,6,7], Diako Ebrahimi[2,3,4], John L. VandeBerg[8], Reuben S. Harris[2,3,4,9], Atsushi Koito[1], Kazuhiko Maeda[6,7,*]

[1]Department of Retrovirology and Self-Defense, Faculty of Life Science, Kumamoto University, Kumamoto 860-8556, Japan
[2]Department of Biochemistry, Molecular Biology, and Biophysics,
[3]Institute for Molecular Virology, [4]Masonic Cancer Center, University of Minnesota, Minneapolis, Minnesota 55455, USA
[5]Department of Immunology, Graduate School of Medical Science, Kumamoto University, Kumamoto 860-8556, Japan
[6]Laboratory of Host Defense, Research Institute for Microbial Diseases,
[7]WPI Immunology Frontier Research Center, Osaka University, Osaka 565-0871, Japan
[8]South Texas Diabetes and Obesity Institute, School of Medicine, The University of Texas Rio Grande Valley, Brownsville/Harlingen/Edinburg, Texas 78520, USA
[9]Howard Hughes Medical Institute, University of Minnesota, Minneapolis, Minnesota 55455, USA

*Correspondence should be addressed to either of these authors (e-mails: tikeda@umn.edu or kazmaeda@biken.osaka-u.ac.jp).

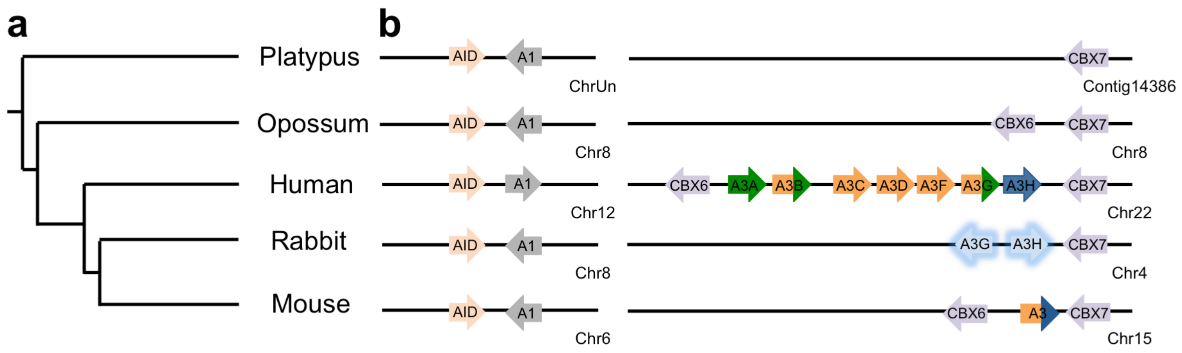

Figure S1. No *A3* gene exists in monotremes and marsupials. (**a**) Phylogenetic tree of selected monotreme (platypus), marsupial (opossum), and eutherian mammals (human, rabbit and mouse). (**b**) Genomic organization of *A1*, *AID*, and *A3* genes. A homology BLAST search was performed for all seven human *A3* genes (*A3A*, *A3B*, *A3C*, *A3D*, *A3F*, *A3G*, and *A3H*) in the genomes of platypus and opossum. Although all species have *A1* and *AID*, non-eutherian mammals did not show any evidence for homologous *A3* genes between the conserved *CBX6* and *CBX7* genes. We also found no evidence to suggest that a gene similar to one of human *A3* genes exists in a different locus in the opossum and platypus genomes. Green, orange, and blue colors indicate Z1, Z2, and Z3 domains of A3 enzymes. For rabbit, two *A3*-like genes (shown in glowing blue color) have been annotated. Based on the available sequences from Ensemble, the rabbit *A3G*-like gene (ensemble ID: ENSOCUG00000023532) appears to encode a single Z1 domain, and the rabbit *A3H*-like gene (ensemble ID: ENSOCUG00000023913) has signatures of Z2 and Z3 domains. However, the database sequences may not be complete or correctly annotated.

```
                  10        20        30        40        50        60        70        80        90       100
         ....|....|....|....|....|....|....|....|....|....|....|....|....|....|....|....|....|....|....|....|
Opossum  MNSKTGPSVGDATLRRRIKPWEFVAFFNPQELRKETCLLYEIKWG-NQNIWRHSNQNTSQHAEINFMEKFTAERHFNSSVRCSITWFLSWSPCWECSKAI
Human    .T.EK...T..P......E....DV.YD.R.....A.........MSRK...S.GK..TN.V.V..IK...S..D.HP.MS..............Q..
Rabbit   .A.EK...NK.Y......E....EV..D.......A.........ASSKT..S.GK..TN.V.V..L..L.S.GRLGP.TC...............M..
Rat      .S.E...VAV.P......E.H..EV..D.R............N..GRHS....TS...NK.V.V..I....T..Y.CPNT.............G...R..
Mouse    .S.E...VAV.P......E.H..EV..D.R............N..GRHSV...TS....N.V.V..L....T..Y.RPNT.............G...R..
                                                                              Catalytic domain
                 110       120       130       140       150       160       170       180       190       200
         ....|....|....|....|....|....|....|....|....|....|....|....|....|....|....|....|....|....|....|....|
Opossum  RKFLDHYPNVTLAIFISRLYWHMDQQHRQGLKELVHSGVTIQIMSYSEYHYCWRNFVDYPQGEEDYWPKYPYLWIMLYVLELHCIILGLPPCLKISGSHS
Human    .E..SRH.G...V.YVA..F.....N....RD..N........RA...YH......N..P.D.AH..Q..P..M...A........S........RRWQ
Rabbit   .E..SQH.G...I..VA..FQ...RRN.....D..T....VRV..V...C...E...N..P.KAAQ..R..PR.MLM.A...Y.............RR.Q
Rat      TE..SR..H...F.Y.A...H.A.PRN....RD.IS........TEQ.SG.......N.SPSN.AH..R..H..VR......Y..........N.LRRKQ
Mouse    TE..SRH.Y...F.Y.A...H.T..RN....RD.IS........TEQ..C.......N..PSN.A...R..H..VK......Y............LRRKQ

                 210       220       230
         ....|....|....|....|....|....|....|.
Opossum  NQLALFSLDLQDCHYQKIPYNVLVATGLVQPFVTWR 235 aa
Human    .H.TF.R.H..N....T..PHI.L....IH.S.A.. 236 aa (67%)
Rabbit   K..TF...TP.Y...KM..PYI.L....L..S.P.. 236 aa (64%)
Rat      P..TF.TIA..S....RL.PHI.W....K------- 229 aa (64%)
Mouse    P..TF.TIT..T....R..PHL.W....K------- 229 aa (66%)
```

Figure S2. Alignment of amino acid sequences of mammalian A1s. Sequences of the predicted full-length mammalian A1 proteins were aligned with Clustal W software. The numbers above each amino acid sequences are the positions of amino acid residues. The identical amino acids to opossum A1 are indicated by a single dot. The amino acid positions that were not aligned are shown with a bar. The amino acid residues involved in the catalytic reaction are shown in red. The amino acid length of opossum A1 is 235 aa. The percent of amino acid identity of each eutherian A1 with opossum A1 is indicated in parentheses at the end of each sequence.

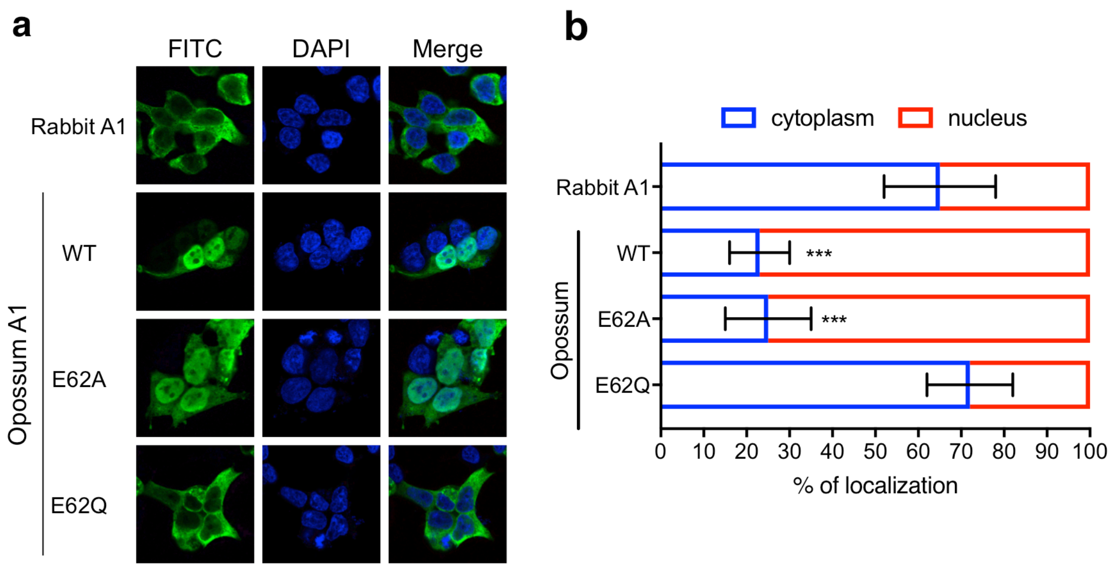

Figure S3. Subcellular localisation of opossum A1 in 293T cells. (**a**) Images show HA-tagged A1s stained with FITC (green) and nuclei stained with DAPI (blue). (**b**) Nuclear and cytoplasmic distribution levels of A1s. The percentage nuclear and cytoplasmic localisation is indicated as the graphs (n = 20, average +/- SD). *P* values represent comparisons with rabbit A1.
